# Adiabatic Quantum-Flux-Parametron: Towards Building Extremely Energy-Efficient Circuits and Systems

**DOI:** 10.1038/s41598-019-46595-w

**Published:** 2019-07-19

**Authors:** Olivia Chen, Ruizhe Cai, Yanzhi Wang, Fei Ke, Taiki Yamae, Ro Saito, Naoki Takeuchi, Nobuyuki Yoshikawa

**Affiliations:** 1 Yokohama National University, Institute of Advanced Sciences, Yokohama, 2408501 Japan; 2 Northeastern University, Department of Electrical and Computer Engineering, Boston, 02115 USA; 3 Yokohama National University, Department of Electrical and Computer Engineering, Yokohama, 2408501 Japan; 4 RESTO, Japan Science and Technology Agency, 4-1-8 Honcho, Kawaguchi, Saitama 332-0012 Japan

**Keywords:** Electrical and electronic engineering, Superconducting devices

## Abstract

Adiabatic Quantum-Flux-Parametron (AQFP) logic is an adiabatic superconductor logic family that has been proposed as a future technology towards building extremely energy-efficient computing systems. In AQFP logic, dynamic energy dissipation can be drastically reduced due to the adiabatic switching operations using AC excitation currents, which serve as both clock signals and power supplies. As a result, AQFP could overcome the power/energy dissipation limitation in conventional superconductor logic families such as rapid-single-flux-quantum (RSFQ). Simulation and experimental results show that AQFP logic can achieve an energy-delay-product (EDP) near quantum limit using practical circuit parameters and available fabrication processes. To shed some light on the design automation and guidelines of AQFP circuits, in this paper we present an automatic synthesis framework for AQFP and perform synthesis on 18 circuits, including 11 ISCAS-85 circuit benchmarks, 6 deep-learning accelerator components, and a 32-bit RISC-V ALU, based on our developed standard cell library of AQFP technology. Synthesis results demonstrate the significant advantage of AQFP technology. We forecast 9,313×, 25,242× and 48,466× energy-per-operation advantage, compared to the synthesis results of TSMC (Taiwan Semiconductor Manufacturing Company) 12 nm fin field-effect transistor (FinFET), 28 nm and 40 nm complementary metal-oxide-semiconductor (CMOS) technology nodes, respectively.

## Introduction

Energy dissipation of Information Communication Technology (ICT), such as data centers, accounts for over 70 billion kiloWatt hours (kWh) of electricity consumption in 2016, or over 2% of the total energy consumption in the U.S.^1^. This number is projected to climb up to 20% of total electricity and emit up to 5.5% of the world’s carbon emissions by 2020. The power dissipation of individual supercomputers is reaching as high as 17,000 kW while the performance is approaching 10 exaFLOPS (EFLOPS)^2^. The significant amount of energy consumption has become a critical problem in modern society, and arouses us of the urgent requirement for energy-efficient computing technologies.

Being widely-known for low energy dissipation and ultra-fast switching speed, Josephson junction-based superconductor logic families have been proposed and implemented to process analog and digital signals for decades. Rapid-Single-Flux-Quantum (RSFQ) logic, proposed by K. Likharev, O. Mukhanov and V. Semenov in 1985^3^, is one leading technology among many alternative superconducting electronic devices. RSFQ-based logic circuits can operate at high clock frequency of hundreds of GigaHertz with very low switching energy on the superconducting devices in the order of 10^−19^ J. However, on-chip resistors are needed to supply a constant DC bias current to the main RSFQ circuit. This will lead to an increasing static power as the circuit scale expands, and makes power dissipation a disadvantage of RSFQ. Various low power technologies, such as energy-efficient single-flux quantum (eSFQ, ERSFQ)^4,5^, reciprocal quantum logic (RQL)^6^, LR-biased RSFQ logic^7^ and low-voltage RSFQ (LV-RSFQ)^8^, have been proposed to (partially) resolve the static power dissipation problem of RSFQ by research groups around the world.

In order to mitigate the power consumption overhead of DC bias, the *Adiabatic Quantum-Flux-Parametron* (AQFP) technology has been proposed using AC bias/excitation currents as both (multi-phase) clock signal and power supply^9^. AQFP circuits operate at a frequency of few GigaHertz, in between conventional CMOS technology and RSFQ logic. The major advantage of AQFP is the remarkable energy efficiency potential. A latest work^10^ analyzed the energy dissipation of an 8-bit AQFP adder and reported a 24*k*_*B*_*T* energy dissipation per junction based on the physical test. This number indicates that AQFP technology is a promising candidate to build low-energy systems approaching Landauder’s limit^11–14^. The demonstrations of several AQFP implementations have been reported, which include an 8-bit carry-look-ahead adder^15^, a 16-word by 1-bit register file^16^, a prototype deep learning accelerator^17^ and a large-scale benchmark chip consisting of 10,000 AQFP logic gates^18^. These results demonstrate the robustness of AQFP technology against circuit parameter variations and the potential towards building very large-scale integrated circuits using AQFP devices. Details of the operation principles of AQFP logic devices can be found in the ref.^9^.

The AQFP technology is promising and rapidly advancing; however, there lacks a systematic, automatic synthesis framework and detailed synthesis results on a large number of benchmark circuits. The framework and results will be beneficial for the further development of AQFP by (i) automatic logic and circuit generation and (ii) illustrating the advantage and limitation of AQFP and the circuit structures that are especially suitable for AQFP technology. In this paper we aim to mitigate this gap, by presenting an automatic synthesis flow and performing synthesis on 18 benchmark circuits, including 11 circuits from the ISCAS-85 benchmark suite, 6 deep-learning accelerator components, and a 32-bit RISC-V ALU. Synthesis is performed using our established standard cell library of AQFP technology (with 4-phase clock signals) and our proposed energy consumption estimations. The proposed energy consumption estimation methodology is accurate and specifically designed for AQFP circuits. Comparison results are presented among our AQFP 10 *kA*/*cm*^2^ standard cell library and TSMC 12 nm FinFET, 28 nm, 40 nm CMOS cell libraries^19–21^. The results demonstrate the consistent energy benefit of AQFP technology. More specifically, it is forecasted that the AQFP technology can achieve a maximum of 9,313×, 25,242× and 48,466× improvements (reduction) in energy consumption per clock cycle, respectively, compared to the results using 12 nm, 28 nm and 40 nm TSMC technologies.

## Results and Discussion

In the experiments, we synthesize 18 circuits, including 11 combinational benchmark circuits from the ISCAS’85 benchmark circuit suite, 6 deep-learning accelerator components, and a 32-bit RISC-V ALU, by using our developed AQFP standard cell library with the 10 *kA*/*cm*^2^ Niobium fabrication technology as well as three semiconductor technologies: TSMC 12 nm, 28 nm and 40 nm. The synthesis methodology on AQFP technology is novel and will be discussed in details next.

### Design flow and energy estimation for AQFP

**Figure 1:**
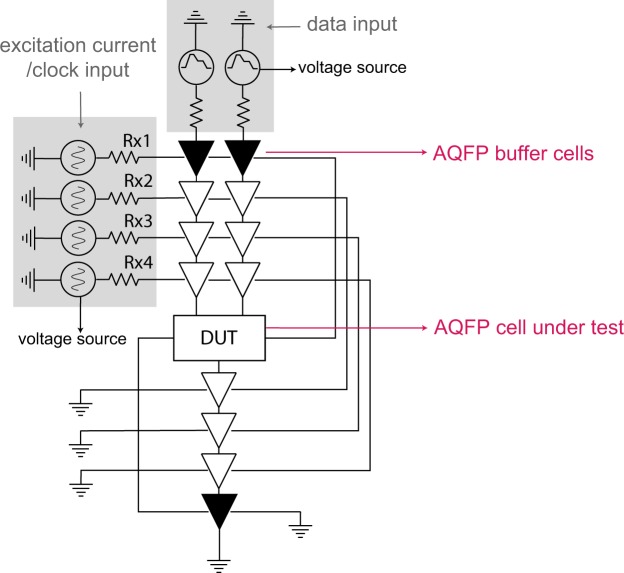
Schematic of a test circuit used for extracting energy dissipation of an under-test AQFP cell.

In this work, we utilize a top-down design flow for AQFP very-large-scale-integration (VLSI) circuits. This design flow starts from high-level synthesis, standard cell library mapping, automatic routing to back-end verification. We adopt the synthesis flow to generate 18 combinational AQFP benchmark circuits as mentioned above. In previous work^22^, we have presented a straightforward energy estimation approach by multiplying the total Josephson-junction count of an AQFP circuit by 5*zJ*. This empirical value 5*zJ* is from the experimental results, showing that the energy dissipating on each AQFP buffer gate using two shunted Josephson junctions is about 10*zJ* at 5 *GHz*^23^, fabricated using the 2.5 *kA*/*cm*^2^ AIST standard process 2 (STP2)^24^. In this study we perform energy estimation in a more accurate way. We carefully extracted the energy dissipation of each cell using the analog simulation tool Jsim^25^ and developed a set of energy models to accurately estimate the energy dissipation of the benchmark circuits using the presented 10 *kA*/*cm*^2^ AQFP cell library. These energy models describe the input-dependent energy dissipation of all gates in the cell library. Figure 1 shows the schematic of a test circuit designed for extracting energy dissipation of an under-test AQFP cell (DUT). The energy extraction flow is summarized in the following steps:

1. The energy dissipation of a single AQFP cell is extremely small. To accurately calculate the energy dissipation of a specific cell, first we insert 4-stage buffers before and after the target cell, and calculate the total energy consumption by integrating the current and voltage of each excitation/clock input for one clock cycle using the following formula:
  1 $$E={\int }_{t}^{t+T}({I}_{x1}{V}_{x1}+{I}_{x2}{V}_{x2}+{I}_{x3}{V}_{x3}+{I}_{x4}{V}_{x4})dt$$
  where *V*_*xn*_ and *I*_*xn*_ are the voltage and current of the excitation lines, generated by Jsim^25^. The summation is over the 4-phase clock signals.
2. Being directly connected to input current source and ground, buffers in the first and last stages (marked in black) are relatively larger than the other buffers (marked in white). Hence, we calculate the energy-delay-product (EDP) of the buffers in the first stage, last stage and middle stage separately.
3. Energy dissipation of the target cell is generated by calculating the difference of the total energy dissipation from step 1 and energy consumption of inserted buffers from step 2, as shown in the following formula. Please note that we use 2 head buffers, 9 middle buffers, and 1 tail buffer, as can be observed from the figure.

2 $${E}_{DUT}={E}_{entire}-\frac{9\times ED{P}_{middle\_buffer}+2\times ED{P}_{head\_buffer}+ED{P}_{tail\_buffer}}{T}$$

Energy dissipation data of all types of cells from AQFP standard cell library have been extracted by the presented method. Table 1 presents the extracted energy dissipation with respect to different data input patterns and different clock rates (frequencies).

**Table 1:** Energy dissipation of buffer, splitter, AND, OR and Majority cells with different input data and clock frequencies.

Test cell	Buffer		Inverter		Splitter 1-to-3		and				or				Majority					
Data pattern	0	1	0	1	0	1	00	01	10	11	00	01	10	11	000	001	010	011	101	111
Freq. (GHz)	Energy dissipation (zJ)																			
1	0.13	0.13	0.22	0.22	0.68	0.68	0.36	9.37	9.37	0.99	0.99	9.37	9.37	0.36	0.13	8.94	9.49	8.94	9.49	0.13
2	0.28	0.28	0.52	0.52	1.66	1.66	0.84	8.77	8.77	1.78	1.78	8.77	8.77	0.84	0.31	8.09	8.56	8.09	8.56	0.31
5	0.83	0.83	2	2	6.17	6.17	3.45	11.59	11.69	5.15	5.15	11.60	11.60	3.45	1.73	9.68	9.79	9.68	9.79	1.73

**Figure 2:**
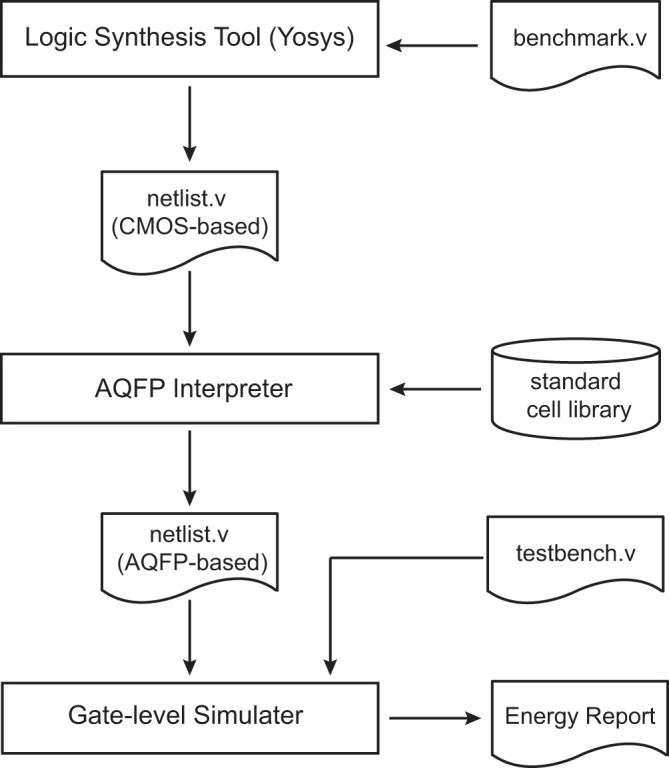
A top-down synthesis flow used for energy estimation of AQFP circuits.

With the developed energy models, we are able to estimate the energy consumption performance of various AQFP benchmark circuits using a top-down synthesis flow. Figure 2 illustrates the details of the synthesis flow.

Logic synthesis in the VLSI design flow plays the role of converting a high-level description of design into an optimized gate-level representation. A CMOS-based open-source synthesis tool ‘Yosys’^26^ is utilized to synthesize circuits described at the behavior level and perform technology mapping to our (AQFP) cell library written in the Liberty library format (.lib). Synthesized circuits are described as structural and-or-inverter (AOI)-based or netlists. Since the AC current serves as clock signal in AQFP circuits, extra AQFP buffers are required to ensure that the dataflow is synchronized at all logic levels of the circuit. In order to make a fair energy estimation, an AQFP *interpreter* is developed to apply post-synthesis to the CMOS logic-based netlist. This interpreter creates an AQFP specific netlist, which is later mapped to our developed energy models to generate statistic files for energy analysis.

### Synthesis and energy estimation for semiconductor technologies

**Figure 3:**
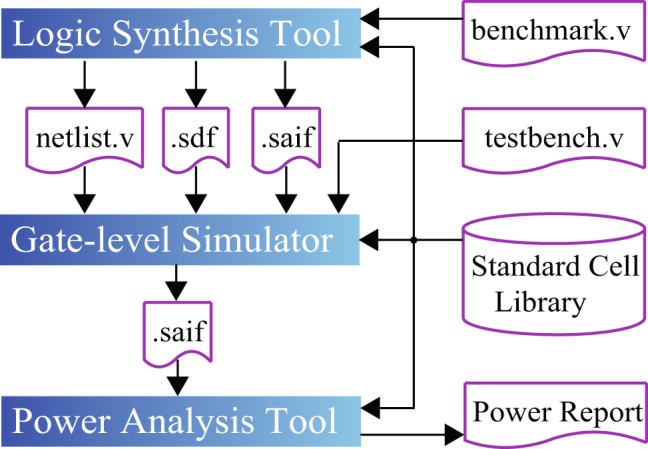
Synthesis and power/energy estimation framework for TSCM 12 nm, 28 nm and 40 nm semiconductor technologies.

For the three representative semiconductor technologies, we adopt the synthesis and power/energy estimation framework described in the paper^27^. We first synthesize a benchmark circuit using the corresponding standard cell library and obtain the synthesized netlist in the Verilog format (named *testbench.v*) and a *standard delay format* (.sdf) file for gate-level simulator, e.g., ModelSim, NC Verilog. For the technology mapping step, we set the target delay of each benchmark circuit to be 30% more than the minimum delay at the given power supply level. A forward-*switching activity interchange format* (.saif) file, which contains state- and path-dependent information of all standard cells, is generated. Meanwhile, based on an input *benchmark.v* file, which specifies the average switching activities at primary inputs of a synthesized circuit, another forward .saif file is generated for the circuit in order to set the primary input activities and produce information of nets in netlist that should be monitored for switching activity to the gate-level simulator. The gate-level simulator determines the information about switching activities at all nets in the netlist and logs it in a backward .saif file. The power/energy analysis tool, e.g., Power Compiler, uses this backward .saif file and the power parameters in standard cell libraries to report accurate power/energy consumption results. The overall synthesis and power/energy estimation flow is depicted in Fig. 3. The TSMC 12 nm and 40 nm library are synthesized at supply voltage *V*_*dd*_ = 0.81 V, whereas the 28 nm library is synthesized at *V*_*dd*_ = 0.72 V. Different from the previous work^27^, the wire capacitances are accounted for in all cases for more accurate results.

### Comparison results and discussions

**Figure 4:**
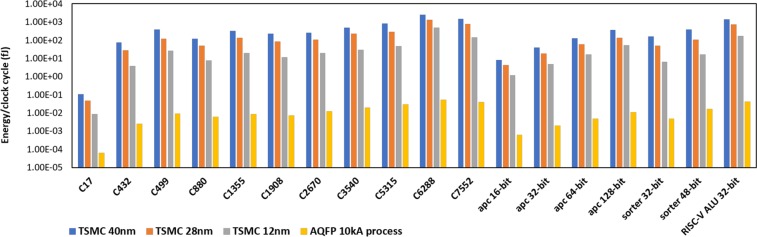
Comparison of energy per clock cycle on different benchmark circuits using TSMC 12 nm, 28 nm 40 nm process and 10 kA AQFP technologies.

**Figure 5:**
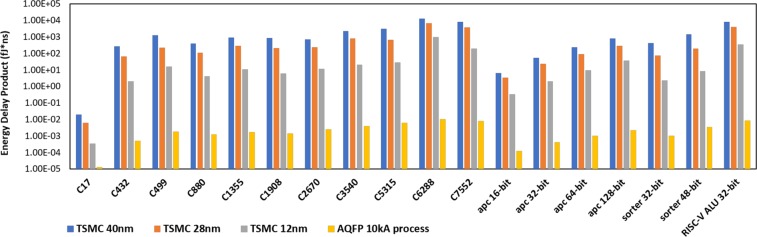
Comparison of energy-delay-product (EDP) on different benchmark circuits using using TSMC 12 nm, 28 nm 40 nm process and 10 kA AQFP technologies.

Table 2 summarizes the power, delay and area of 18 synthesized benchmark circuits using TSMC 12 nm FinFET, 28 nm and 40 nm CMOS libraries. Table 3 summarizes the energy dissipation results among AQFP and three semiconductor technologies in terms of energy-per-clock cycle (EPC) and energy-delay-product (EDP). Comparisons of EPC and EDP between semiconductor and superconductor technologies are further illustrated in Figs 4 and 5. For AQFP circuits, we do not report the end-to-end delay because each gate in AQFP is essentially pipelined, and therefore, the end-to-end delay is very much different compared with the *initialisation interval* of the input instructions. The initialisation interval equals to the clock period and is utilised for EDP calculation for AQFP circuits. Buffers and splitters are accounted for in the AQFP synthesis results. From the comparison results, one could conclude that the AQFP circuits consistently outperform the semiconductor counterparts in terms of both EPC and EDP. The maximum gains on EPC and EDP are 4.84 × 10^4^ and 1.18 × 10^5^, respectively, compared with 40 nm TSMC library. The EDP gains are in general higher than EPC because the clock frequency in AQFP can reach as high as 5 GHz, which is higher compared with that in the CMOS circuits and systems.

**Table 2:** Summary of benchmark synthesis results using different semiconductor (FinFET/CMOS) logic families, in terms of power, delay and area.

Benchmark	TSMC 12 nm (FinFET)			TSMC 28 nm (CMOS)			TSMC 40 nm (CMOS)		
	Power (*μW*)	Delay (*ns*)	Area (*μm*^2^)	Power (*μW*)	Delay (*μW*)	Area (*μm*^2^)	Power (*μW*)	Delay (*ns*)	Area (*μm*^2^)
c17	0.21409	0.04	0.88	0.36157	0.13	1.86	0.59578	0.18	3.63
c432	7.0583	0.54	20.23	11.138	2.45	41.55	21.023	3.52	74.39
c499	44.583	0.6	50.04	63.443	1.89	114.16	123	3.22	214.78
c880	15.266	0.51	41.33	22.395	2.21	86.72	37.665	3.23	158.31
c1355	37.407	0.54	50.14	66.136	2.09	109.46	118.5	2.8	205.93
c1908	21.881	0.53	37.23	34.547	2.42	80.56	59.311	3.78	143.79
c2670	32.304	0.6	70.82	51.803	2.1	155.33	88.996	2.83	269.89
c3540	42.421	0.7	90.59	66.772	3.42	195.12	116	4.33	334.08
c5315	78.802	0.59	165.47	134.3	2.22	363.09	223.2	3.74	615.54
c6288	245.6	2.01	284.64	270.30	4.95	576.53	528.60	4.86	1036.93
c7552	105.9	1.35	189.2	172.5	4.61	423.26	294.9	5.15	760.01
apc16	4.3308	0.28	7.74	5.3552	0.8	15.98	10.077	0.81	29.26
apc32	12.273	0.4	18.62	15.565	1.22	38.22	28.943	1.38	68.72
apc64	30.407	0.55	41.06	38.405	1.52	84.28	71.783	1.78	151.5
apc128	74.916	0.7	87.83	87.761	1.91	178.36	164.8	2.17	317.52
sorter32	18.449	0.35	81.7	34.141	1.49	175.13	61.967	2.57	307.77
sorter48	33.828	0.49	160.96	60.835	1.81	330.75	109.8	3.57	597.62
alu32	81.577	2.08	191.6	136.3	5.36	426.59	244.1	5.78	721.68

**Table 3:** Summary of benchmark synthesis results using different technologies of semiconductor (FinFET/CMOS) and superconductor (AQFP) logic families.

	TSMC 12 nm		TSMC 28 nm		TSMC 40 nm		AQFP 10 kA process	
	EPC (fJ)	EDP (fJ · ns)	EPC (fJ)	EDP (fJ · ns)	EPC (fJ)	EDP (fJ · ns)	EPC (fJ)	EDP (fJ · ns)
C17	0.0085636	0.000342544	0.0470041	0.006110533	0.1072404	0.019303272	0.000063849	1.27698E-05
C432	3.811482	2.05820028	27.2881	66.855845	74.00096	260.4833792	0.002484509	0.000496902
C499	26.7498	16.04988	119.90727	226.6247403	396.06	1275.3132	0.009260182	0.001852036
C880	7.78566	3.9706866	49.49295	109.3794195	121.65795	392.9551785	0.006266488	0.001253298
C1355	20.19978	10.9078812	138.22424	288.8886616	331.8	929.04	0.00864267	0.001728534
C1908	11.59693	6.1463729	83.60374	202.3210508	224.19558	847.4592924	0.007243422	0.001448684
C2670	19.3824	11.62944	108.7863	228.45123	251.85868	712.7600644	0.012586774	0.002517355
C3540	29.6947	20.78629	228.36024	780.9920208	502.28	2174.8724	0.019773314	0.003954663
C5315	46.49318	27.4309762	298.146	661.88412	834.768	3122.03232	0.030201986	0.006040397
C6288	493.656	992.24856	1337.985	6623.02575	2568.996	12485.32056	0.053005104	0.010601021
C7552	142.965	193.00275	795.225	3665.98725	1518.735	7821.48525	0.039949245	0.007989849
apc 16-bit	1.212624	0.33953472	4.28416	3.427328	8.16237	6.6115197	0.000611122	0.000122224
apc 32-bit	4.9092	1.96368	18.9893	23.166946	39.94134	55.1190492	0.002017221	0.000403444
apc 64-bit	16.72385	9.1981175	58.3756	88.730912	127.77374	227.4372572	0.00494974	0.000989948
apc 128-bit	52.4412	36.70884	135.46148	279.0506488	357.616	776.02672	0.01110811	0.002221622
sorter 32-bit	6.45715	2.2600025	50.87009	75.7964341	159.25519	409.2858383	0.004992	0.0009984
sorter 48-bit	16.57572	8.1221028	110.11135	199.3015435	391.986	1399.39002	0.01694828	0.003389656
RISC-V ALU 32-bit	169.68016	352.9347328	730.568	3915.84448	1410.898	8154.99044	0.043076487	0.008615297

One common concern about the superconducting technology and applications is the energy overhead for cooling. It is widely estimated that the cooling energy is about 400×^28^ compared with the energy dissipation of superconducting circuits. Of course this value depends on environmental temperature. For space applications the cooling energy can be significantly reduced. Even when the cooling energy is accounted for, AQFP technology will still enable around two orders of magnitude reduction (improvement) in energy dissipation or EDP, compared with semiconductor counterparts. This is unique characteristics of AQFP and cannot be achieved using RSFQ logics requiring DC bias currents. These results demonstrate the potential of AQFP technology and applications for large-scale, high-performance, and energy-efficient computations.

**Figure 6:**
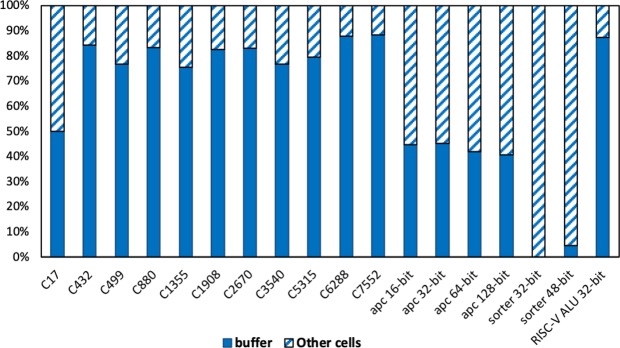
Proportion of inserted buffers with different total cell counts in 11 benchmark circuits using AQFP technology.

Another concern about superconducting circuits is the overhead of inserted buffers and splitters. Especially for large-scale AQFP circuits, will the inserted buffers and splitters account for a significant portion of Josephson junction (JJ) counts and energy dissipations? We illustrate in Table 4 and Fig. 6 the proportion of different cells including splitters and buffers. One could observe that splitters only account for a very small portion of the total JJ counts. This is because AQFP supports up to 1-to-4 splitters, which is more flexible compared with 1-to-2 splitters in RSFQ. Buffers, on the other hand, account for larger portion of JJ counts in larger circuit benchmarks compared with smaller ones. The reason is because larger circuits have more paths and are more delay-balanced compared with smaller ones. The results demonstrate that the overhead of buffers will be reduced for larger-scale circuits, which is a good news for the future development of superconducting technology and applications. For the self-path-balanced circuits such as the approximate-parallel-counters and bitonic sorters, which are commonly used in the stochastic-computing based deep-learning accelerator^17^, the total JJ count can be further optimized. In addition, the experimental results of APCs show that AQFP circuits can benefit from majorty-based logic synthesis, as the three-input majority gates utilize the same JJ resources as two-input AND/OR gates in AQFP.

**Table 4:** Josephson-junction counts and proportion of different cells in 18 benchmark circuits using AQFP technology.

Benchmark	JJ counts	AND	INV	OR	SPL	MAJ	buffer	total cell counts	Estimated Area (*μm*^2^)
C17	60	4	0	2	3	0	9	18	23
C432	2958	47	3	72	72	0	1047	1241	1109
C499	7256	169	8	238	236	0	2163	2814	2721
C880	6804	153	11	150	151	0	2331	2796	2552
C1355	6662	190	0	203	230	0	1922	2545	2498
C1908	8000	182	14	174	206	0	2712	3288	3000
C2670	13802	277	216	246	246	0	4870	5855	5176
C3540	15104	476	31	415	415	0	4433	5770	5664
C5315	25506	740	106	571	641	0	8073	10131	9565
C6288	71884	1427	215	639	1544	0	27985	31810	26957
C7552	46480	873	191	552	758	0	18016	20390	17430
apc 16-bit	304	10	0	7	23	12	42	94	114
apc 32-bit	912	38	0	33	63	28	126	278	342
apc 64-bit	2134	98	0	57	153	61	266	635	800
apc 128-bit	4652	222	0	127	335	128	560	1372	1745
sorter 32-bit	3840	240	0	240	480	0	0	960	1440
sorter 48-bit	7040	320	0	480	800	80	80	1760	2640
RISC-V ALU 32-bit	47396	1040	36	477	1059	0	18052	20644	17774

We further estimated the chip area that required for the synthesized circuits using 10 *kA*/*cm*^2^ superconducting process with a minimum wire width of 50 *nm*. Considering the superconductor fabrication is still at an early development stage, the integration density is about 4× to 26× lower when comparing to the TSMC 40 nm CMOS technology. In the future, these numbers can be further reduced by introducing multi-layer techniques^29^ and transformer-free AQFP technique^30^.

In a nutshell, the synthesis results of AQFP circuits are highly promising in terms of energy-efficient and high-performance computing. With the future advancing and maturity of AQFP fabrication technology, we will anticipate broader applications ranging from space applications and large-scale computing facilities such as data centers.

## Methods

### AQFP standard cell library

**Figure 7:**
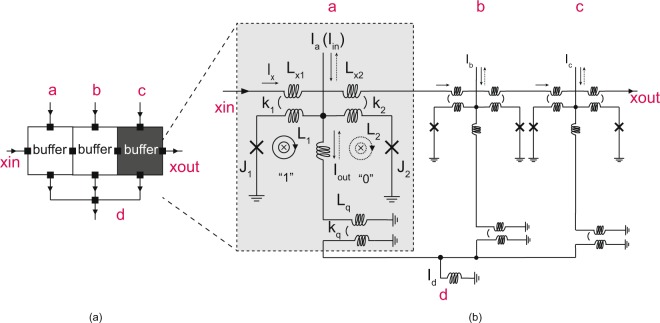
(**a**) Symbol of an AQFP majority gate, which consists of 3 buffers and a 3-to-1 merger (branch); (**b**) Junction-level schematic of an AQFP majority gate.

The basic logic structure of AQFP circuits is a buffer consisting of a double-Josephson-junction SQUID^31^, as shown in the shaded area of Fig. 7(b). An AQFP logic gate is basically driven by AC-power, which serves as both excitation current and power supply.

As shown in the shaded area of Fig. 7(b), excitation fluxes are applied to the superconducting loops via inductors *L*_1_, *L*_2_, *L*_*x*1_ and *L*_*x*2_ by applying the excitation current *I*_*x*_, which is usually in the order of hundreds of micro-amperes (e.g. ±800 *μ*A). One single flux quantum is either stored in the left or right loop, depending on the small input current *I*_*in*_ with a typical value of several micro-amperes (e.g. ±5 *μ*A). As a result, the device can work as a buffer cell and the logic state can be represented by the direction of the output current *I*_*out*_. In this way, the existence of a quantum flux either in the left or the right loop can be encoded as logic ‘1’ or ‘0’.

Furthermore, the AQFP inverter and constant cells (constant ‘1’ or ‘0’ cells) are designed from the AQFP buffer. The AQFP inverter is designed by negating the coupling coefficient of the output transformer in an AQFP buffer, whereas the AQFP constant gates are created by introducing asymmetry in the inductors for excitation fluxes in the standard AQFP buffer design. The key characteristic of AQFP logic is that different type of gates are built from AQFP buffer. This characteristic offers effective design methodology for a standard cell library and ensures the robustness with respect to circuit parameters, as long as the AQFP buffer is carefully designed in terms of the symbolic view and the physical layout.

**Figure 8:**
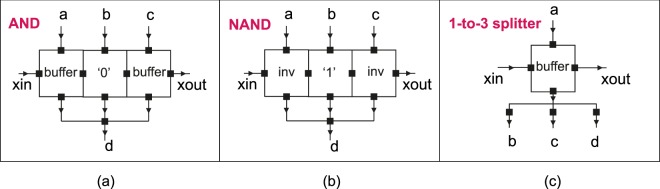
(**a**) Symbol of an AQFP AND gate, which consists of 2 buffers, a constant ‘0’ and a 3-to-1 merger (branch); (**b**) Symbol of an AQFP AND gate, which consists of 2 inverters, a constant ‘1’ and a 3-to-1 merger (branch); (**c**) Symbol of an AQFP 1-to-3 splitter, which consists of a buffer and a 1-to-3 branch.

With the presented basic building blocks (buffer, inverter, constant ‘0’ and constant ‘1’ gates), it is very effective to build an AQFP standard cell library by introducing the *minimalist design approach*^15^, i.e., designing more complicated gates using a bottom-up manner. For instance, majority gates are designed by merging the outputs of three buffers through a 3-to-1 branch as shown in Fig. 7. More examples on AND, NAND, and splitter are presented in Fig. 8. As an example, the AQFP AND gate is implemented similar to the majority gate with one of the three AQFP buffers replaced by a constant ‘0’ gate.

With our proposed minimalist design methodology, standard cell libraries have been built for two major 10 *kA*/*cm*^2^ Niobium processes, the high-speed standard process (HSTP)^32^ and the MIT-LL SFQ process^33^. Both cell libraries contain the same basic cells: BUFFER, INVERTER, AND, OR, MAJORITY, SPLITTER and off-chip INTERFACE, and they are designed with the optimized circuit parameters to achieve the best performance.

**Figure 9:**
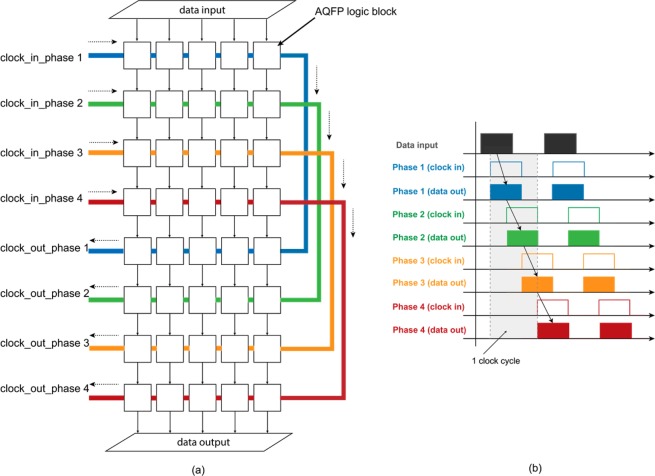
(**a**) A typical meander structure of AQFP circuits using 4-phase clocking scheme; (**b**) Illustration of data propagation between clock phases.

Different to the conventional CMOS technology, both combinational and sequential logic cells in AQFP cell libraries are driven by AC-power. The AC power serves as clock signal as well to synchronize the outputs of all gates in the same clock phase. As a result, data propagation in AQFP circuits is achieved by exploiting the overlapping of different clock signals in the neighboring phases. Figure 9 shows the meander structure of a typical clocking scheme of AQFP circuits (a) and an illustration of how data propagates between clock phases. The hardware-description-language (HDL) SystemVerilog^34^ is employed to build logical models for each individual logic cell, which will be further used for logic synthesis, timing analysis, and circuit verification^35^. These HDL models specify the input/output pins, logic functions, timing parameters and fabrication process. The presented HDL models are written in a parameterized approach, and can be easily modified for different fabrication technologies and low-level circuit parameters.

## Supplementary information

Figure S1

## References

[1] Shehabi, A. *et al*. United states data center energy usage report. *Tech. Rep*. LBNL-1005775, Lawrence Berkeley National Laboratory (2016).

[2] Strohmaier, E., Dongarra, J., Simon, H. & Meuer, M. Top500 supercomputer sites. https://www.top500.org/.

[3] Likharev KK, Semenov VK (1991). Rsfq logic/memory family: a new Josephson-junction technology for sub-terahertz-clock-frequency digital systems. IEEE Transactions on Applied Superconductivity.

[4] Mukhanov OA (2011). Energy-efficient single flux quantum technology. IEEE Transactions on Applied Superconductivity.

[5] Kirichenko DE, Sarwana S, Kirichenko AF (2011). Zero static power dissipation biasing of rsfq circuits. IEEE Transactions on Applied Superconductivity.

[6] Herr QP, Herr AY, Oberg OT, Ioannidis AG (2011). Ultra-low-power superconductor logic. Journal of applied physics.

[7] Yoshikawa N, Kato Y (1999). Reduction of power consumption of rsfq circuits by inductance-load biasing. Superconductor Science and Technology.

[8] Tanaka M, Ito M, Kitayama A, Kouketsu T, Fujimaki A (2012). 18-ghz, 4.0-aj/bit operation of ultra-low-energy rapid single-flux-quantum shift registers. Japanese Journal of Applied Physics.

[9] Takeuchi N, Ozawa D, Yamanashi Y, Yoshikawa N (2013). An adiabatic quantum flux parametron as an ultra-low-power logic device. Superconductor Science and Technology.

[10] Takeuchi N, Yamae T, Ayala C, Suzuki H, Yoshikawa N (2019). An adiabatic superconductor 8-bit adder with 24kbt energy dissipation per junction. Applied Physics Letters.

[11] Landauer R (1961). Irreversibility and heat generation in the computing process. IBM Journal of Research and Development.

[12] Anderson NG (2018). Landauer’s limit and the physicality of information. The European Physical Journal B.

[13] Chiuchiù D, Diamantini M, Gammaitoni L (2015). Conditional entropy and landauer principle. EPL (Europhysics Letters).

[14] Bérut A (2012). Experimental verification of landauer’s principle linking information and thermodynamics. Nature.

[15] Takeuchi N, Yamanashi Y, Yoshikawa N (2015). Adiabatic quantum-flux-parametron cell library adopting minimalist design. Journal of Applied Physics.

[16] Tsuji N (2017). Design and implementation of a 16-word by 1-bit register file using adiabatic quantum flux parametron logic. IEEE Transactions on Applied Superconductivity.

[17] Chen, O., Ma, X., Wang, Y., Takeuchi, N. & Yoshikawa, N. Design of adiabatic-quantum-flux-parametron register files using a top-down design flow. In *2018 Applied Superconductivity Conference (ASC 2018)* (IEEE, Seattle, 2018).

[18] Narama, T., Yamanashi, Y., Takeuchi, N., Ortlepp, T. & Yoshikawa, N. Demonstration of 10k gate-scale adiabatic-quantum-flux-parametron circuits. In *15th International Superconductive Electronics Conference (ISEC. 2015)*, 1–3, 10.1109/ISEC.2015.7383438 (IEEE, 2015).

[19] Taiwan Semiconductor Manufacturing Company. Tsmc 16/12 nm technology, https://www.tsmc.com/english/dedicatedFoundry/technology/16nm.htm.

[20] Taiwan Semiconductor Manufacturing Company. Tsmc 28 nm technology, https://www.tsmc.com/english/dedicatedFoundry/technology/28nm.htm.

[21] Taiwan Semiconductor Manufacturing Company. Tsmc 40 nm technology, https://www.tsmc.com/english/dedicatedFoundry/technology/40nm.htm.

[22] Xu, Q., Yamanashi, Y., Takeuchi, N., Ayala, C. & Yoshikawa, N. Performance analysis of synthesized benchmark circuits implemented in adiabatic superconductor logic. In *16th International Superconductive Electronics Conference (ISEC. 2017)*, 1–3 (IEEE, 2017).

[23] Takeuchi N, Yamanashi Y, Yoshikawa N (2013). Measurement of 10 zj energy dissipation of adiabatic quantum-flux-parametron logic using a superconducting resonator. Applied Physics Letters.

[24] Nagasawa S, Hashimoto Y, Numata H, Tahara S (1995). A 380 ps, 9.5 mw josephson 4-kbit ram operated at a high bit yield. IEEE Transactions on Applied Superconductivity.

[25] Fang, E. S. & Duzer, T. V. A josephson integrated circuit simulator (jsim) for superconductive electronics application. *Int. Superconductivity Electronics Conf*. 407–410 (1989).

[26] Wolf, C. Yosys, http://www.clifford.at/yosys/.

[27] Xie, Q. *et al*. 5 nm finfet standard cell library optimization and circuit synthesis in near-and super-threshold voltage regimes. In *2014 IEEE Computer Society Annual Symposium on VLSI*, 424–429, 10.1109/ISVLSI.2014.101 (2014).

[28] Holmes DS, Ripple AL, Manheimer MA (2013). Energy-efficient superconducting computing’power budgets and requirements. IEEE Transactions on Applied Superconductivity.

[29] Ando T (2017). Three-dimensional adiabatic quantum-flux-parametron fabricated using a double-active-layered niobium process. Superconductor Science and Technology.

[30] Arai K, Takeuchi N, Yamashita T, Yoshikawa N (2019). Adiabatic quantum-flux-parametron with pi josephson junctions. Journal of Applied Physics.

[31] Clarke John, Braginski Alex I. (2004). The SQUID Handbook.

[32] Nagasawa S., Hinode K., Satoh T., Akaike H., Kitagawa Y., Hidaka M. (2005). Reliability evaluation of Nb 10 kA/cm2 fabrication process for large-scale SFQ circuits. Physica C: Superconductivity and its Applications.

[33] Tolpygo, S. K. *et al*. Advanced Fabrication Processes for Superconducting Very Large Scale Integrated Circuits. *ArXiv e-prints* (2015).

[34] Ieee standard for systemverilog–unified hardware design, specification, and verification language. *IEEE Std 1800-2017 (Revision of IEEE Std 1800-2012)* 1–1315, 10.1109/IEEESTD.2018.8299595 (2018).

[35] Xu Q, Ayala CL, Takeuchi N, Yamanashi Y, Yoshikawa N (2016). Hdl-based modeling approach for digital simulation of adiabatic quantum flux parametron logic. IEEE Transactions on Applied Superconductivity.

