# Supplementary material for: Adiabatic Quantum-Flux-Parametron: Towards Building Extremely Energy-Efficient Circuits and Systems
Source: Sci Rep. 2019 Jul 19;9:10514. doi: 10.1038/s41598-019-46595-w (PMC6642163; doi:10.1038/s41598-019-46595-w)
Supplement: Supplementary file 1 — Figure S1 [file 41598_2019_46595_MOESM1_ESM.pdf]

Supplementary Materials for

Adiabatic Quantum-Flux-Parametron: Towards Building Extremely Energy-Efficient Circuits and Systems

Olivia Chen, Ruizhe Cai, Yanzhi Wang, Fei Ke, Taiki Yamae, Ro Saito, Naoki Takeuchi and Nobuyuki Yoshikawa

This file includes:

Figure S1

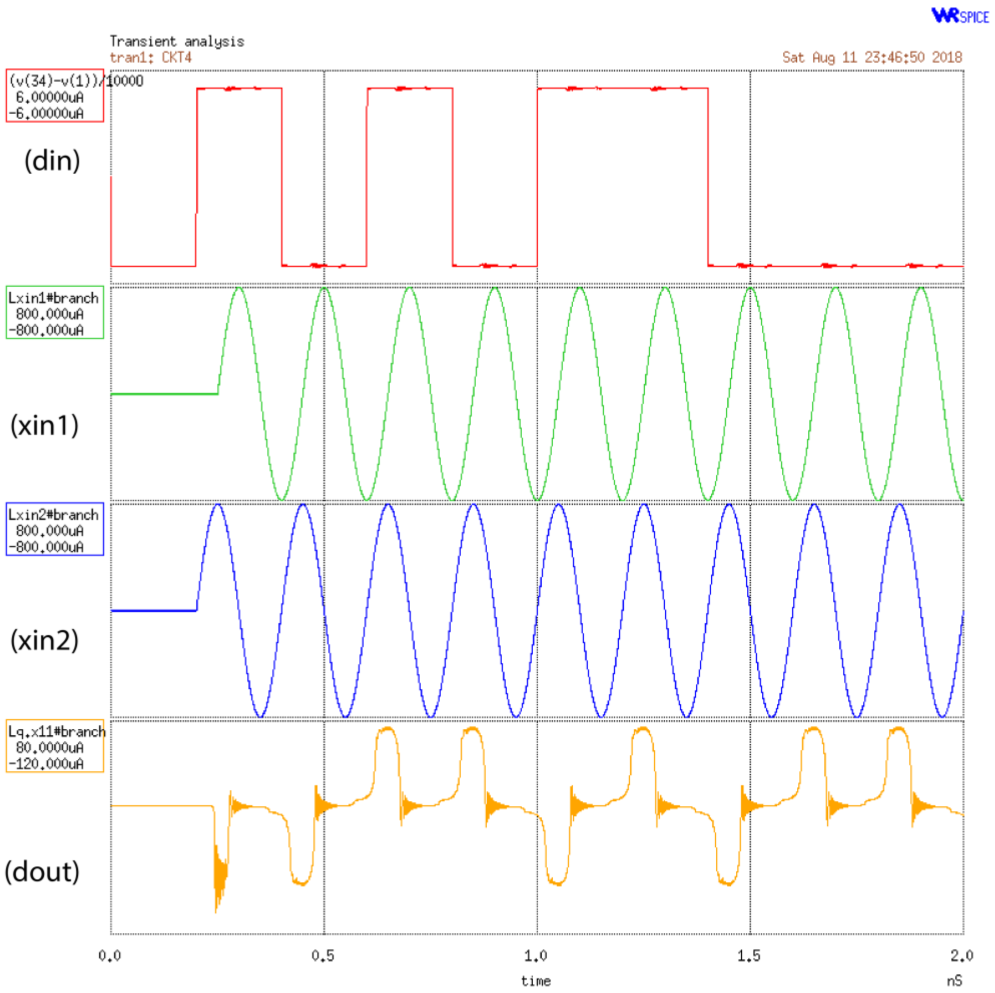

Figure S1. Simulation waveform of a 12-stage AQFP buffer chain at 5 GHz. Signals from top to bottom: buffer chain input (din: input of the first buffer) as 101011, AC source 1 (xin1: generates phase 1 and 3), AC source 2 (xin2: generates phase 2 and 4), and the output of the final (12th) buffer (dout) in the buffer chain as 001101011 with three random initial outputs 001. This is because of the meander structure of AQFP circuits. In a 12-stage AQFP circuit, it takes three clock cycles to propagate the data to the output. Input peak-to-peak amplitude is ±5 μA, AC amplitude is 800 μA, and DC is set to 1.2 mA.
